# Psychometric Properties of the Diabetes Eating Problem Survey—Revised in Arab Adolescents with Type 1 Diabetes: A Cross-Cultural Validation Study

**DOI:** 10.3390/bs15081026

**Published:** 2025-07-29

**Authors:** Abdullah M. Alguwaihes, Shuliweeh Alenezi, Renad Almutawa, Rema Almutawa, Elaf Almusahel, Metib S. Alotaibi, Mohammed E. Al-Sofiani, Abdulmajeed AlSubaihin

**Affiliations:** 1 Division of Endocrinology, Department of Internal Medicine, College of Medicine, King Saud University, Riyadh 11461, Saudi Arabia; moh.alsofiani@gmail.com; 2 Diabetes Center, Dallah Hospital, Riyadh 12381, Saudi Arabia; 3 Department of Psychiatry, College of Medicine, King Saud University, Riyadh 11451, Saudi Arabia; 4 SABIC Psychological Health Research and Applications Chair (SPHRAC), Department of Psychiatry, College of Medicine, King Saud University, Riyadh 11451, Saudi Arabia; 5 Department of Internal Medicine, King Fahad Medical City, Riyadh 11525, Saudi Arabia; rabalmutawa@kfmc.med.sa; 6 Department of Neurology, National Neurosciences Institute, King Fahad Medical City, Riyadh 11525, Saudi Arabia; raalmutawa@kfmc.med.sa (R.A.); ealmusayhil@kfmc.med.sa (E.A.); 7 University Diabetes Center, King Saud University Medical City, King Saud University, Riyadh 11472, Saudi Arabia; ametib@ksu.edu.sa; 8 Division of Endocrinology, Department of Pediatrics, College of Medicine, King Saud University, Riyadh 11461, Saudi Arabia; asubaihin@ksu.edu.sa

**Keywords:** type 1 diabetes mellitus, eating disorders, DEPS-R, adolescents, cross-cultural validation

## Abstract

Objectives: The objective of this manuscript is to translate, adapt, and validate an Arabic version of the Diabetes Eating Problem Survey—Revised (DEPS-R) questionnaire to assess disordered eating behaviors (DEBs) in adolescents with T1D in Saudi Arabia. Additionally, the study sought to estimate the prevalence of DEBs and analyze its associations with glycemic control and diabetes-related complications. Methods: A cross-cultural validation study was conducted following the COSMIN guidelines. The DEPS-R questionnaire was translated into Arabic through forward and backward translation involving expert panels, including psychiatrists, diabetologists, and linguists. A sample of 409 people with type 1 diabetes (PwT1D) (58.4% females) aged 12–20 years was recruited from outpatient diabetes clinics in the five main regions of Saudi Arabia. Participants completed the Arabic DEPS-R and the validated Arabic version of the SCOFF questionnaire. Sociodemographic, anthropometric, and biochemical data were collected, and statistical analyses, including confirmatory factor analysis (CFA) and internal consistency tests, were conducted. Results: The Arabic DEPS-R exhibits strong internal consistency (Cronbach’s alpha = 0.829) and high test–retest reliability (ICC = 0.861), with a CFA supporting a three-factor structure, namely body weight perception, disordered eating behaviors (DEBs), and bulimic tendencies. Notably, higher DEPS-R scores are significantly linked to elevated HbA1c levels, increased BMI, and more frequent insulin use. Alarmingly, 52.8% of participants show high-risk DEB, which is directly associated with poor glycemic control (HbA1c ≥ 8.1%) and a heightened risk of diabetic ketoacidosis (DKA). Conclusions: The Arabic DEPS-R is a valid and reliable tool for screening DEBs among Saudi adolescents with T1D. Findings underscore the necessity for early identification and intervention to mitigate the impact of EDs on diabetes management and overall health outcomes.

## 1. Introduction

According to the Diabetes Atlas (10th edition), 35,000 children and adolescents in Saudi Arabia suffer from type 1 diabetes (T1D) (T1D; International Diabetes Federation, 2021). Saudi Arabia now among the top 10 countries in incidence of T1D (Ogle et al., 2025). In a recent systematic review involving 14 studies (N = 9076) ascertaining the different eating disorders (EDs) in the T1D population (N = 1391) versus nondiabetic individuals (N = 7688), patients with type 1 diabetes (PwT1D) are at a substantially higher risk of eating disorders (ED) and disordered eating behavior (DEB), particularly binge eating and bulimia nervosa, than individuals without T1D (Dean et al., 2024). Furthermore, the SEARCH for Diabetes in Youth study estimated that 21.2% of all PwT1D have DEBs (Nip et al., 2019). Among the multiple risk factors identified in the literature are diet and carbohydrate intake monitoring, insulin-related weight gain, and associated body dissatisfaction, all contributing to the increasing incidence of EDs among PwT1D (Dean et al., 2024; Nip et al., 2019). Moreover, DEBs and EDs have been associated with poor glycemic control secondary to behaviors, such as skipping breakfast, binge eating, self-induced vomiting, and insulin omission (Tarçın et al., 2023). Mortality was also found to be higher in PwT1D with DEBs, including insulin restriction, than those without DEBs (Goebel-Fabbri et al., 2008). Such behaviors are associated with an increased risk of diabetes-related complications and decreased life expectancy (Goebel-Fabbri et al., 2008). Therefore, taking precautions and designing an approach to identifying these unhealthy behaviors early on is essential to improving the prognosis among vulnerable populations as those with PwT1D. Additionally, adolescence is a critical developmental stage characterized by rapid physical, psychological, and social changes that impact T1D management. Pubertal changes can alter insulin sensitivity, complicating glycemic control (Shpitzer et al., 2021).

Psychologically, adolescents develop executive functions, such as impulse control and decision making, which are essential for adhering to T1D regimens but are often immature, leading to risk-taking behaviors, like insulin omission (Casey et al., 2011). Socially, peer influence and the pursuit of autonomy can exacerbate body image concerns, increasing the risk of DEBs (Daniel et al., 2023). These factors underscore the need for targeted screening tools, like the Arabic DEPS-R, to identify DEBs in this vulnerable population.

Scales are the most common screening tools used to identify EDs. Recently, significant efforts have been made to validate these scales in Middle Eastern countries, the earliest of which was the Arabic version of the Eating Attitude Test (EAT-26), validated among Saudi female students (Al-Subaie et al., 1996). Among the most widely used scales is the five-question SCOFF questionnaire, which addresses the core features of anorexia nervosa and bulimia nervosa. The SCOFF questionnaire was translated into Arabic in 2015 (A-SCOFF) and has since been instrumental in screening for EDs (Aoun et al., 2015). Another widely used screening tool is the Eating Disorder Examination Questionnaire (EDE-Q), which was validated in Saudi Arabia in 2023 after being translated into Arabic (Aldubayan et al., 2023). However, both the A-SCOFF and EDE-Q are designed for the general population and are not specific to individuals with diabetes. This warranted the development of a unique scale specifically designed to screen for DEBs in PwT1D, as EDs arising in this population may present in distinct ways compared to the general population.

To effectively address disordered eating behaviors in individuals with T1D, the Diabetes Eating Problem Survey (DEPS) was originally developed for adults (Markowitz et al., 2010). It was later refined into the Diabetes Eating Problem Survey—Revised (DEPS-R) to better assess contemporary youth with T1D (Markowitz et al., 2010). The DEPS-R, a 16-item tool derived from the original 28-item DEPS, has demonstrated strong psychometric properties, including excellent internal consistency (Cronbach’s α = 0.86) and robust external validity (Markowitz et al., 2010). Given its reliability, DEPS-R has been recommended for routine clinical use, particularly in identifying adolescents with T1D who may be at risk for eating disorders. Additionally, the tool has been translated into multiple languages, including the recently published Arabic version, which has shown good construct validity and reliability comparable to the original version (Hummadi et al., 2023). However, the validation of the Arabic DEPS-R was based on data from a single center with a limited sample size, raising concerns about its generalizability to the broader and diverse Saudi population. Further research involving larger, more representative samples across different regions is essential to ensure its cultural applicability and clinical utility in Saudi Arabia.

This study has the following two primary objectives: (1) to translate, adapt, and validate an Arabic version of the Diabetes Eating Problem Survey—Revised (DEPS-R) for screening disordered eating behaviors (DEBs) among Saudi adolescents with type 1 diabetes (T1D), and (2) to estimate the prevalence of DEBs and its associations with glycemic control and diabetes-related complications. The secondary objective is to evaluate the psychometric properties of the Arabic DEPS-R, including its internal consistency, test–retest reliability, and construct validity, to ensure its cultural applicability. We hypothesize that the Arabic DEPS-R will demonstrate strong psychometric properties comparable to the original English version and that higher DEPS-R scores will be associated with poorer glycemic control (HbA1c ≥ 8.1%) and increased risk of complications, such as diabetic ketoacidosis (DKA). By validating a culturally tailored screening tool, this study aims to contribute to clinical practice by enabling early identification of DEBs in Saudi adolescents with T1D, facilitating timely interventions to improve diabetes management and reduce the risk of adverse health outcomes.

## 2. Materials and Methods

This is a cross-cultural adaptation study to translate and validate the Arabic version of the DEPS-R questionnaire conducted at the Endocrinology Unit of the Department of Internal Medicine, College of Medicine in King Saud University (KSU), Riyadh, Saudi Arabia, from September 2021 to September 2023. The COSMIN standard for validating the cross-cultural validity of a patient-reported outcome measure (PROM) was followed throughout this research (COSMIN, n.d.). Permission from the original authors of the DEPS questionnaire was obtained before conducting this study, and the Institutional Review Board (IRB) approval from the College of Medicine in KSU, Riyadh, Saudi Arabia, was granted in February 2022 [No. E-22-6560].

### 2.1. Translation

Forward translation was conducted by two native Arabic speakers, one of whom was an Arabic-speaking psychiatrist ESA, alongside a professional Arabic-speaking English translator with a PhD in English language and translation ERA. Both independently created a version of the DEPS-R in Arabic, followed by a meeting to resolve differences between the original and translated versions and merge them into one single version. A third native Arabic speaker, a diabetologist, was consulted to resolve differences in translated statements if an agreement with the original translators was not reached.

Two native English speakers with advanced proficiency in Arabic performed the back translation of the DEPS-R (AMF and ZAM). Both had previous experience with Arabic translation. The provided back translations were then evaluated by an expert group composed of ten diabetologists and psychiatrists (MA, SA, MA, RS, and AA), who reviewed semantic equivalence and resolved any uncertainties and differences in meaning and appropriateness of terminology. Since no items were deleted or added during the translation phases, the final Arabic version contained the same number of items as the original version.

### 2.2. Participants

A total of 409 adolescents with T1D (239 females and 170 males) aged 12–20 years were recruited from outpatient diabetes clinics at KSU Medical City (KSUMC) and through online platforms. For online recruitment, a link was distributed via targeted private groups on Telegram, WhatsApp, and X (formerly Twitter), accessible only to individuals with T1D, and shared with diabetes educators and physicians for snowball sampling across Saudi Arabia’s five main regions. Participants were included if they self-reported a confirmed T1D diagnosis and agreed to participate. To support the reliability of self-reported diagnoses, all participants confirmed being on insulin therapy (86.1% using injections, 13.9% using insulin pumps), with 85.8% reporting hypoglycemic events (≥1 per week) and 98% using glucose monitoring (77.5% via CGM devices, like Libre/Dexcom/Medtronic, 20.5% via glucometers). These characteristics, combined with the age group (12–20 years) and reported comorbidities (e.g., 23% with a sibling with T1D, 8.9% with kidney disease, 34.4% with thyroid dysfunction), align with the clinical profile of T1D, reducing the likelihood of false reporting. However, reliance on self-reported diagnoses remains a limitation, as noted in Section 4.2. Exclusion criteria included age <12 years or >20 years. All participants provided informed consent.

Sociodemographic data, including age, family social status, household monthly income, residence region, comorbid conditions, treatment modalities, and diabetes history, were recorded directly from the participants. Anthropometric and biochemical data were retrieved from electronic medical records. Participants were then asked to fill out the Arabic DEPS-R questionnaire, followed by the SCOFF questionnaire, which had already been translated and validated in Arabic, with permission obtained from the translator.

### 2.3. Data Analysis

The SPSS IBM statistical software version 21, the standalone Factor Analysis program (FACTOR-9.2, Lorenzo-Seva & Ferrando, 2013), and the AMOS SPSS IBM software version 20 were used for statistical computing and analysis. Mean and standard deviation (SD) were used to describe continuously measured variables; the median and the interquartile range were used to describe non-normally distributed continuous variables. The frequency and percentage (%) were used to describe categorical variables. The Kolmogorov–Smirnov statistical test and histograms were used to assess normality. The Cronbach’s α test was used to evaluate the internal consistency of the measured questionnaire, and the inter-class correlations (ICC) coefficient test was used to assess the test–retest reliability of the DEPS questionnaire; the Spearman’s correlations test assessed correlations between metric-measured variables. Coefficient (R) values of 0.10–0.29 were interpreted as small, 0.30–0.49 as medium, and 0.50–1.0 as significant (Cohen, 1988). Exploratory factor analysis (EFA) was used to evaluate the dimensionality of the DEPS questionnaire. Structural equation modeling-based confirmatory factor analysis (CFA) with the maximum likelihood method was used to confirm a three-factor latent measurement model for the DEPS questionnaire. Concurrent validity was measured by comparing DEPS-R with the SCOFF questionnaire (Morgan et al., 2000). The area under the receiver operating curve (AUC ROC) was used to assess the specificity and sensitivity of the DEPS scale score at predicting SCOFF scale dichotomized eating problem levels. 

Confirmatory factor analysis (CFA) was selected to test the hypothesized factor structure of the Arabic DEPS-R and to assess the model fit based on theory and previous findings from the original version. Receiver operating characteristic (ROC) analysis was used to evaluate the diagnostic performance of the DEPS-R in identifying individuals at risk of disordered eating behaviors, providing sensitivity, specificity, and optimal cutoff points. These tools were chosen for their robustness and relevance in validating screening instruments. Multivariable linear regression analysis was applied to assess the significance of the predictors for diabetic adolescents’ mean perceived eating problems (DEPS) score. The association between the analyzed predictor variables with the adolescents’ mean DEPS score was expressed by Beta coefficients (β) with 95% confidence limits. Significance was set at *p* < 0.05.

## 3. Results

### 3.1. PwT1D Characteristics

A total of 409 PwT1D participated in the study by completing a questionnaire. Sociodemographic characteristics revealed that 58.4% of participants were female, with an average age of 15.7 years (SD = 2.8). The mean age at diabetes diagnosis for the sample of adolescents was 9.4 years (SD = 4.6 years). BMI classification showed that 9% of participants were underweight, 64.3% were normal weight, 16.1% were overweight, and 10.5% were obese. The majority of participants (81.4%) reported that both parents are alive and living together. A smaller proportion (7.3%) indicated that both parents are alive but separated. Additionally, 5.6% reported that either one or both parents are deceased. Household monthly income varied, with 24.9% not disclosing their income, 15.6% earning less than 5000 SAR/month, 20.5% earning between 5000–9999 SAR/month, 14.2% earning 10,000–14,999 SAR/month, and 24.7% earning 15,000 SAR/month or more. Geographically, most participants (50.4%) resided in the Central Region of Saudi Arabia, followed by the Western (16.6%), Eastern (9.8%), Northern (7.3%), and Southern (5.9%) regions (Table 1).

Table 2 shows the participants’ medical history and diabetes-related outcomes and factors. The findings showed that 23% had at least one sibling with T1D. The results also showed that 8.1% were previously diagnosed with depression, 14.7% had an anxiety disorder, and 23% reported other comorbidities. These comorbidities included 8.9% with kidney disease, 46.7% with celiac/gluten disease, 6.7% with asthma or allergic airway disease, 34.4% with thyroid dysfunction, 6.7% with adrenal insufficiency, and 17.8% with other less frequent conditions. Around 27% of the participants reported their last HbA1c as being >9%, 21.8% reported 8.1–9%, 21.5% reported 7.1–8%, and 22.5% reported HbA1c ≤7%. Regarding blood glucose monitoring methods, 2% reported that they were not monitoring their blood glucose levels, 20.5% used conventional glucometers, and 77.5% used devices, such as Libre, Dexcom, or Medtronic, to monitor their daily blood glucose levels. Most (86.1%) used insulin injections for their daily required insulin, while 13.9% used insulin pumps. Those who were prescribed insulin therapy reported their average daily insulin intake. On average, they received 23.5 ± 11.3 units of long-acting insulin, 21.9 ± 14.8 units of short-acting insulin, and 11.3 ± 8.6 units of premixed insulin. When asked about the frequency of hypoglycemic events (blood glucose levels < 70 mg/dL) per week, 14.2% had no hypoglycemic events, 35.9% experienced 1–2 events/week, 31.3% experienced 3–4 events/week, 13.2% experienced 5–7 events/week, and 5.4% experienced ≥8 events/week. Additionally, they were asked about hospital admissions due to diabetic ketoacidosis (DKA) in the past six months. The results showed that 74.3% had not been hospitalized due to DKA, 18.8% had been admitted once, 4.6% were admitted twice, and 2.2% were admitted three or more times within the last six months.

### 3.2. Internal Consistency and Reliability

Table 3 presents the internal structure evidence and item–total scale corrected correlations for the Arabic version of the DEPS-R scale. The 16-item Diabetes Eating Problem Survey (DEPS-R) demonstrated strong internal consistency among Arabic-speaking children, with an estimated Cronbach’s α = 0.829, reflecting the reliability of the scores. Additionally, test–retest reliability was assessed using the intraclass correlation coefficient (ICC), and the results indicated a high level of stability in the score estimates over time (ICC = 0.861, n = 41).

### 3.3. Construct Validity

**Figure 1:**
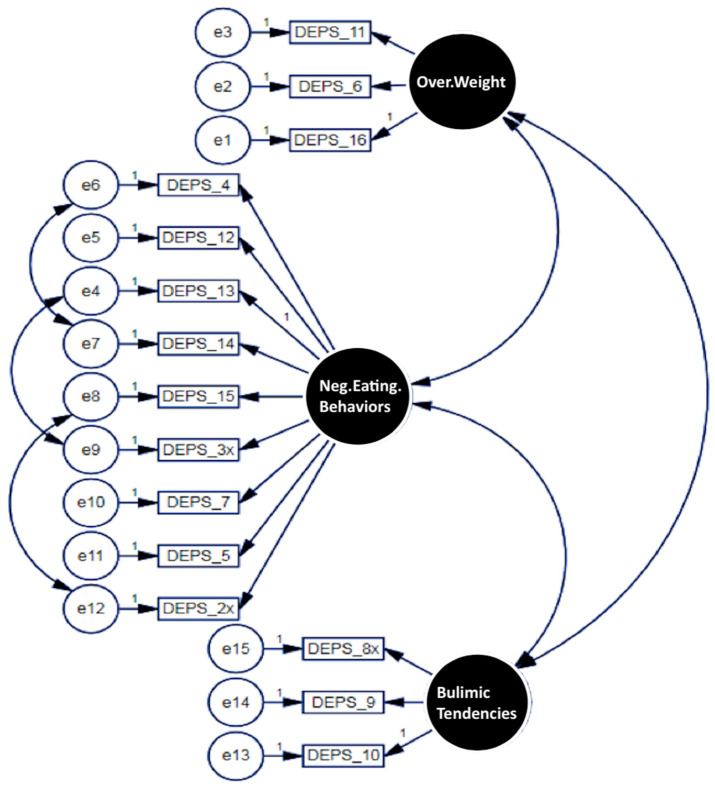
Identified domains.

To assess the structure of the DEPS-R scale, EFA followed by CFA was applied to the 15 items comprising the questionnaire (Table 4). EFA findings identified 3 latent factors within the covariance matrix of the 15 items. Sampling adequacy (KMO = 0.862) indicated an appropriate sample size. At the same time, the determinant index (0.023) and Bartlett’s test (χ^2^ (91) = 1511.2, *p* < 0.001) confirmed the suitability of factor analysis for the Arabic DEPS-R scale. The resulting three-factor solution was evaluated using SEM CFA. As presented in Table 4 and Figure 1, the items characterizing adolescents with T1D perceptions of body weight loaded significantly onto the first latent factor, labeled “Problematic Body Weight Perception”. Items measuring negative eating behaviors and habits markedly loaded onto the second factor, labeled “Negative Eating Behaviors”. Finally, items capturing bulimic behaviors (e.g., overeating, deliberate vomiting, and overeating to elevate blood sugar) loaded significantly onto the third latent factor, labeled “Bulimic Tendencies”. The finalized 3-factor solution for the Arabic version of the DEPS-R questionnaire was accepted. Subscale scores for each latent factor were computed by averaging the responses to the items within each factor and multiplying the resulting mean score by 16, yielding a total score ranging from 0 to 80 points. Higher scores indicated worse perceptions of the constructs.

### 3.4. Concurrent Validity and Sensitivity Analysis

A bivariate Spearman’s correlations test (Table 5) suggested that the overall mean perceived DEPS-R scale score correlated positively and significantly with its three subscale scores (*p* < 0.01 for each). Additionally, the mean perceived SCOFF total score correlated positively and significantly with their overall DEPS-R scale score (r = 0.665, *p* < 0.01). The analysis also showed that the overall DEPS-R scale score correlated positively with their standardized BMI (r = 0.266, *p* < 0.01). Similarly, HbA1c levels correlated positively and significantly with their overall DEPS-R score (r = 0.30, *p* < 0.01). Furthermore, the children’s total daily intake of long-acting insulin units correlated positively, though weakly, with their overall DEPS-R scale score (r = 0.182, *p* < 0.01). Mean perceived body weight subscale score correlated positively with mean perceived negative eating behaviors subscale score (r = 0.506) and perceived bulimic behaviors subscale score (r = 0.31, *p* < 0.01 for both). Overall SCOFF score correlated positively and significantly with mean perceived body weight score (r = 0.568), BMI, recent HbA1c levels, and daily intake of long-acting insulin units (*p* < 0.01 for each). Mean perceived negative eating behaviors subscale score correlated positively and significantly with perceived bulimic behaviors subscale score (r = 0.342, *p* < 0.01). Moreover, there was a positive correlation between age and SCOFF scores (r = 0.229, *p* < 0.01). BMI and HbA1c levels also correlated positively with SCOFF scores (*p* < 0.01 for each). Finally, AUC showed that the DEPS-R scale had strong predictive power for identifying children at risk of eating disorders (SCOFF) (0.816; *p* < 0.001).

### 3.5. Predictors

Multivariable linear regression analysis of adolescents’ average perceived DEPS-R score (Table 6) showed that sex and age were not significantly correlated with the DEPS-R score. However, the body mass index (BMI) score was significantly and positively correlated with the DEPS-R score. For each additional one-unit increase in BMI, the model predicted an average increase of 0.351 points in the DEPS-R score, while accounting for other predictor variables (*p* < 0.001). The analysis also indicated that those with a HbA1c level of ≥8.1% had significantly higher DEPS-R scores than those with HbA1c < 8.1% (β coefficient = 1.654, *p* < 0.001). PwT1D who required hospital admission due to DKA in the past six months had significantly higher DEPS-R scores than those who had not been hospitalized (β coefficient = 2.302, *p* = 0.03). Additionally, those with a prior diagnosis of depression had significantly higher DEPS-R scores than those without depression (β coefficient = 6.106, *p* = 0.003). Anxiety was not correlated with DEPS-R scores (*p* = 0.58). Lastly, the SCOFF scores significantly correlated with DEPS-R score (β coefficient = 6.121, *p* < 0.001).

### 3.6. Adolescents’ Perceptions 

Analysis of the adolescents’ perceptions of eating problems and eating disorders revealed some important findings (Table 7). The mean perceived diabetes problem eating (DEPS) score was 22.68/80 points, with 47.2% exhibiting low problematic eating behavior and 52.8% showing high problematic eating behavior (DEPS-R score ≥ 20 points). These findings have significant implications for adolescent health. The mean perceived obesity subscale score was 20.5/80 points, and the negative eating behavior subscale score was 28.61/80 points. The mean perceived bulimic behaviors subscale score for the DEPS scale was 2.41/80 points.

## 4. Discussion

In this study, we recognized the importance of presenting the validation results and offering a comprehensive analysis of the underlying factor structure. Understanding these component dimensions significantly enhances the clinical relevance of the screening tool and supports a more accurate interpretation of the disordered eating behaviors being assessed. The Diabetes Eating Problem Survey (DEPS) was initially crafted to assess adults, but it has since evolved into the Diabetes Eating Problem Survey—Revised (DEPS-R). This updated version is designed to more effectively evaluate disordered eating behaviors among today’s youth living with type 1 Diabetes (T1D) (Markowitz et al., 2010).

In recent years, the DEPS-R has garnered significant attention from researchers worldwide, who have explored its factor structure in adult T1D populations across different languages. This collaborative effort has yielded valuable insights. For instance, Sancanuto et al. identified five key factors through exploratory factor analysis, namely food attitudes, bulimic behavior, weight control, avoidance, and restriction (Sancanuto et al., 2017).

Meanwhile, Karastogiannidou et al. supported a single-factor model, and Wisting et al. proposed a three-component structure that highlights maladaptive eating habits, a fixation on thinness or weight, and the concerning practice of manipulating blood glucose levels for weight management (Karastogiannidou et al., 2021; Wisting et al., 2013). Pinna et al. echoed Wisting’s findings, affirming the importance of these identified components (Pinna et al., 2017). Most recently, a 2024 evaluation of the DEPS-R’s psychometric properties in an adult Dutch population found that the single-factor solution showed robust internal consistency, while the three-factor solution demonstrated good internal consistency within the individual factors (Embaye et al., 2024). Our study brings exciting news, as we identified three distinct latent factors that characterize disordered eating behaviors in adolescents with T1D.

The first factor, “Perceptions of Body Weight/Obesity”, highlights concerns about body image and weight, emphasizing the psychological effects of insulin-related weight gain. The second factor, “Negative Eating Behaviors or Habits”, encompasses maladaptive behaviors, like restricted food intake and compensatory actions that aim to manage both weight and glycemic control. Finally, the third factor, “Bulimic Habits”, includes problematic behaviors, such as binge eating, self-induced vomiting, and insulin manipulation for blood sugar management. Remarkably, these findings are in line with studies by Wisting et al. and Embaye et al., despite their focus on adult populations, showcasing the relevance of these issues across age groups (Wisting et al., 2013; Embaye et al., 2024).

An important aspect of our research was the validation of the Arabic DEPS-R scale, which adds a significant contribution to the growing literature on culturally relevant screening tools. While the A-SCOFF and EDE-Q scales have been validated for Arabic-speaking populations, they do not specifically cater to the unique experiences of individuals with diabetes (Aoun et al., 2015; Aldubayan et al., 2023). Our findings confidently demonstrate that the DEPS-R is a reliable instrument for identifying disordered eating behaviors in those with T1D, boasting strong internal consistency (Cronbach’s α = 0.829) and impressive test–retest reliability (ICC = 0.861). These results are promising and align with previous DEPS-R validation studies conducted in Western populations(Markowitz et al., 2010). However, the slight discrepancies in item–total correlations suggest that cultural modifications may be needed to enhance sensitivity to Saudi adolescents’ experiences. These differences may stem from cultural perceptions of body image and food-related behaviors unique to Middle Eastern societies (Al-Subaie et al., 1996).

Looking at the secondary objective of our study, we found that a significant 52.8% of PwT1D displayed disordered eating behaviors (DEBs), as measured by the Arabic version of the DEPS-R. This statistic is notably higher than the 21.2% reported in the SEARCH for Diabetes in Youth study, which suggests that regional and cultural factors, as well as variations in screening tools, may contribute to this discrepancy (Nip et al., 2019). Our findings contribute to ongoing discussions about the heightened risk of disordered eating behaviors in PwT1D, reinforcing the critical need for awareness and intervention. A systematic review by Dean et al. corroborates this, indicating an increased risk of eating disorders among PwT1D (Dean et al., 2024). The high prevalence of DEBs (52.8%) in our study reflects the unique challenges of adolescence, where the developing prefrontal cortex contributes to difficulties in self-regulation, such as inconsistent insulin administration or dietary adherence (Salah et al., 2022). Social pressures, including body image concerns amplified by media and peer influences, may further drive DEBs, as evidenced by the significant correlation between DEPS-R scores and BMI (r = 0.266, *p* < 0.01). Family dynamics also play a role, with 23% of participants having a sibling with T1D, potentially influencing eating behaviors through shared dietary practices or emotional support (Hilliard et al., 2018). Interventions, like cognitive-behavioral therapy, which target self-regulation skills, have shown promise in improving both DEBs and glycemic outcomes in adolescents with T1D (Stadler et al., 2025). Our results reflect these trends, illustrating a concerning connection between insulin omission and binge eating, both of which can lead to poor glycemic control.

Moreover, our study highlights that the risk of DEBs tends to increase with age among adolescents with T1D. This aligns with other research indicating that older adolescents often face greater concerns regarding body weight and engage in unhealthy eating practices (Daniel et al., 2023). We also found a notable relationship between DEPS-R scores and the method of insulin administration. Those using insulin injections demonstrated greater body dissatisfaction compared to individuals utilizing insulin pumps. The significant association between DEPS-R scores and insulin administration methods in our study echoes previous research that indicates that PwT1D using insulin injections are more likely to experience body dissatisfaction compared to those using insulin pumps, and that continuous insulin delivery methods, such as pumps, may reduce body image concerns and improve adherence to diabetes management plans (Falcão & Francisco, 2017).

Another vital area of focus in our study was the intersection between DEBs and psychological comorbidities. We observed that 8.1% of participants had a prior diagnosis of depression, while 14.7% were diagnosed with anxiety disorders. These findings agree with previous research indicating that adolescents with T1D and DEBs frequently exhibit heightened levels of anxiety and depressive symptoms (Kara et al., 2023). These findings illuminate the complex relationship between mental health and eating patterns in adolescents with T1D and underline the necessity for integrated care approaches that address both physical and emotional well-being.

The association between DEBs and glycemic control in our study is particularly noteworthy. Our results revealed that adolescents with higher DEPS-R scores had significantly elevated HbA1c levels, an outcome that has been supported by the prior literature (Sildorf et al., 2018). This relationship highlights the importance of integrating mental health and nutritional counseling into diabetes management strategies. A randomized clinical trial by Stadler and colleagues emphasized the effectiveness of multidisciplinary approaches that incorporate psychological therapy alongside traditional diabetes care to address disordered eating behaviors and improve metabolic outcomes (Stadler et al., 2025). Moreover, our study found that 23% of participants had a sibling diagnosed with T1D. This is consistent with genetic studies that indicate a familial predisposition to both T1D and DEBs (Salah et al., 2022). The presence of a sibling with T1D may influence eating behaviors, either through shared dietary restrictions or increased awareness of diabetes-related complications. Future research should further explore the role of family dynamics in the development and management of DEBs in PwT1D.

### 4.1. Practical Implication

This study emphasizes the importance of developing culturally relevant tools, such as the Arabic DEPS-R, to screen for disordered eating behaviors—particularly in diverse populations. Our work highlights the urgent need for support systems tailored to the unique challenges faced by individuals living with type 1 diabetes (T1D). While the DEPS scale can serve as a general screening tool, and the total score may help identify individuals at risk, our findings suggest that the scale is not unidimensional. Therefore, relying solely on the total or mean score may not be sufficient for diagnosing diabetic eating disorders. Instead, the subscale scores may provide more meaningful and nuanced insights into the various dimensions of disordered eating behaviors in this population. By fostering awareness and promoting open dialogue, we hope to contribute to better health outcomes and an improved quality of life for those navigating both diabetes and complex eating behaviors.

### 4.2. Limitations and Prospective Directions

The authors acknowledge some limitations. The present Arabic version of DEPS-R was validated together with the Arabic version of the SCOFF questionnaire, which is the most widely used tool for screening eating disorders in adolescents. Other common tools for eating disorders, such as EATS-26, have already been validated with the Arabic DEPS-R (Aoun et al., 2015). Given the inherent limitation of DEPS-R as a screening tool and not a diagnostic tool for EDs, the obtained prevalence of DEBs in the present study is only suggestive and further investigations are still necessary to confirm diagnosis. Another limitation is relying on self-reporting of complications and diagnoses of depression and anxiety.

## 5. Conclusions

The present study contributes to the literature by validating the Arabic DEPS-R and demonstrating its effectiveness in screening for DEBs among Saudi adolescents with T1D. The high prevalence of high-risk DEBs underscores the need for routine screening and early intervention strategies to improve health outcomes in this vulnerable population. Future research should focus on longitudinal studies to explore the impact of DEBs on diabetes management and assess interventions tailored to address both psychological and metabolic concerns in PwT1D.

## Figures and Tables

**Table 1:** Sociodemographic characteristics of participants (N = 409).

	Frequency	Percentage
**Sex**		
Female	239	58.4
Male	170	41.6
**Age (years), mean (SD)**	15.7 (2.8)	
**Age at diagnosis (years), mean (SD)**	9.4 (4.6)	
**Age group (years)**		
12–14	168	41.1
15–17	110	26.9
18–20	131	32
**Height (cm), mean (SD)**	155.6 (17.7)	
**Weight (kg), mean (SD)**	55.2 (17.6)	
**Body mass index (BMI) kg/m^2^, mean (SD)**	22.25 (5.3)	
**BMI level**		
Underweight	37	9
Normal	263	64.3
Overweight	66	16.1
Obese	43	10.5
**Family social status**		
Both parents are alive and bonded	333	81.4
Both parents are alive and separated	30	7.3
Either or both parents are deceased	23	5.6
**Households Monthly Income (SAR/month)**		
Do not know/prefer not to disclose	102	24.9
<5000	64	15.6
5000–9999	84	20.5
10,000–14,999	58	14.2
≥15,000	101	24.7
**Residence region**		
Eastern	40	9.8
Western	68	16.6
Northern	30	7.3
Southern	24	5.9
Central	247	60.4

**Table 2:** Participants’ medical history and diabetes-related outcomes.

	Frequency	Percentage
**Siblings with T1D**	94	23
**Depression**	33	8.1
**Anxiety**	60	14.7
**Other comorbidities**	109	26.6
**Comorbidities**		
Kidney disease	8	8.9
Celiac disease/gluten disease	42	46.7
Asthma/allergic airways	6	6.7
Thyroid disease	31	34.4
Adrenal glands disease	6	6.7
Others	16	17.8
**Recent (last month) HbA1c (%)**		
Unknown	28	6.8
≤7	92	22.5
7.1–8	88	21.5
8.1–9	89	21.8
≥9.1	112	27.4
**Method to monitor your blood glucose level**		
I do not monitor	8	2
Using conventional BG monitoring (glucometer) devices	84	20.5
Using Libre/Dexcom/Medtronic devices and methods	317	77.5
**How do you take your daily insulin?**		
Injections	352	86.1
Pump	57	13.9
**How many times per week do you experience hypoglycemic events?**		
None	58	14.2
1–2	147	35.9
3–4	128	31.3
5–7	54	13.2
≥8	22	5.4
**Times diagnosed with diabetic ketoacidosis (DKA) in the last 6 months**		
None	304	74.3
Once	77	18.8
Twice	19	4.6
Three or more times	9	2.2
**How many long-acting insulin units do you take/day, mean (SD)**		23.5 (11.3)
**How many short acting insulin units do you take/day, mean (SD)**		21.9 (14.8)
**How many insulin units do you take/day, mean (SD)**		11.3 (8.6)

**Table 3:** Internal consistency and reliability.

Scale	No. of Items	Cronbach’s α
DEPS—R	16	0.829
SCOFF	5	0.759
Test–retest internal consistency	ICC = 0.861, n = 41	

**Table 4:** CFA yielded item–factor standardized regression coefficients (i.e., loadings).

Latent Factor	Items Loading to Factors	Regression Estimate	*p*-Value
**Problematic Body Weight Perception**			
16) I would rather be thin than to have good control of my diabetes.		0.459	<0.001
6) I feel that it is difficult to lose weight and control my diabetes at the same time.		0.612	<0.001
11) I feel fat when I take all of my insulin.		0.593	<0.001
**Negative Eating Behaviors**			
13) After I overeat, I skip my next insulin dose.		0.607	<0.001
12) Other people tell me to take better care of my diabetes.		0.533	<0.001
4) When I overeat, I do not take enough insulin to cover the food.		0.588	<0.001
14) I feel that my eating is out of control.		0.789	<0.001
15) I alternate between eating very little and eating huge amounts.		0.695	<0.001
3) Other people have told me that my eating is out of control.		0.675	<0.001
7) I avoid checking my blood sugar when I feel like it is out of range.		0.510	<0.001
5) I eat more when I am alone than when I am with others.		0.567	<0.001
2) I skip meals and/or snacks.		0.310	<0.001
**Bulimic Tendencies**			
10) I try to eat to the point of spilling ketones in my urine.		0.604	<0.001
9) I try to keep my blood sugar high so that I will lose weight.		0.754	<0.001
8) I make myself vomit.		0.300	<0.001
One item was removed (item DEPS-1: Losing weight is my essential goal.).			

**Table 5:** Bivariate correlations between adolescents’ measured eating problems perception and other relevant factors.

DEPS Score	DEPS Score	OB	NEB	BB	SCOFF	Age	BMI	Hba1c	LAI	SAI
Diabetes eating problem score-1										
Obesity (OB) perception score	0.742 **									
Negative Eating Behaviors (NEB) score	0.932 **	0.506 **								
Bulimia Behaviors (BB) score	0.433 **	0.310 **	0.342 **							
SCOFF score	0.665 **	0.568 **	0.569 **	0.371 **						
Age (years)	0.138 **	0.088	0.093	0.094	0.229 **					
BMI Score	0.266 **	0.311 **	0.131 **	0.015	0.255 **	0.394 **				
Recent Hba1c Level	0.300 **	0.178 **	0.329 **	0.208 **	0.170 **	−0.064	0.008			
No. of long-acting (LAI) insulin units/day	0.182 **	0.121 *	0.147 **	0.082	0.205 **	0.222 **	0.365 **	0.212 **		
No. of short-acting (SAI) insulin units taken/day	0.044	0.009	0.047	−0.027	0.023	0.115 *	0.183 **	0.043	0.329 **	
No. of premixed insulin units taken per day	0.166	0.333	0.086	0.164	0.456	0.078	−0.046	−0.439	0.041	−0.316

* *p*-value < 0.05, ** *p*-value < 0.01.

**Table 6:** Multivariable linear regression analysis of diabetic adolescents’ mean perceived DEPS scale score (N = 409).

	Unstandardized β Coefficient	95% C.I. for β Coefficient		*p*-Value
		Upper	Lower	
Constant	3.07	−3.21	9.35	0.34
Male	0.95	−0.88	2.79	0.31
Age (years)	−0.12	−0.47	0.23	0.50
BMI score	0.35	0.17	0.53	<0.001
Most recent Hba1c level ≥ 8.1%	1.64	0.91	2.38	<0.001
Use of insulin pump	−2.38	−5.02	0.27	0.078
No. of hypoglycemic episodes per week	0.28	−0.04	0.59	0.08
Positive history of DKA last 6 months	2.30	0.18	4.42	0.03
Positive history of depression	6.11	2.08	10.14	0.003
Positive history of anxiety disorder	0.86	−2.24	3.96	0.58
Mean perceived SCOFF score	6.12	5.26	6.98	<0.001

Note: Dependent outcome variable = Adolescents’ mean perceived diabetes problem eating (DEPS) score. Model overall significance: f (10,398) = 42.21, *p*-value<0.001, R-squared = 0.515, Adj. R-squared = 0.503.

**Table 7:** Descriptive analysis of the diabetic adolescents’ overall eating problems perceptions.

	Mean	SD
Diabetes Eating Problem (DEPS) scale total score	22.68	12.82
Problematic Eating Behavior (DEPS score > 20 points)		
No	193	47.2
Yes	216	52.8
DEPS.Factor1 Obesity perception score	20.5	19.6
DEPS.Factor2 Negative Eating Behaviors (NEB) score	28.61	16.5
DEPS.Factor3 Bulimia Behaviors (BB) score	2.41	7.1
Useful Eating Disorder screening questions (SCOFF) questionnaire	1.24	1.14
SCOFF score ≥ 3 points		
No	345	84.4
Yes	64	15.6

## Data Availability

The raw data supporting the conclusions of this article will be made available by the authors upon request.
